# Abstracts and Gleanings

**Published:** 1882-11-20

**Authors:** 


### ABSTRACTS AND GLEANINGS.

**Extirpation of the Spleen.**—In an article on extirpation of the spleen, written by Dr. D. J. Zesas, of Zurich, and published in von Langenbeck's Archiv (28 Band, 1 Heft., p. 157), the author prefaces a report of a number of experiments made by himself on animals, with a review of similar experiments made from time to time by other observers. His results were far more satisfactory than those of his predecessors, which he attributes to thefact that their experiments were not confined solely to extirpation of the spleen, but included as well extirpation of the thyroid gland, and, in one of the cases, section of the left vagus nerve.

Zesas operated on six rabbits, all of which recovered rapidly from the operation; five of them were killed at times varying from one to seventeen weeks afterwards, and the post-mortem revealed no marked pathological change.

The author was able to find thirty cases reported of the operation performed on man, with the following results: In twenty cases there was prolapse of the organ, resultant upon penetrating wounds of the abdomen. All of these recovered. In seven cases the operation was performed for the cure of diseased spleen; three times successfully. The remaining three cases are added to the report: one of them terminated, in all probability, favorably (Volney Howard, for hypertrophy following malarial disease); the other two fatally.

After mentioning the modes of operation, as advised and carried out by Shultze, Adelmann and Pean, Zesas concludes his paper with a summary of the contra-indications for the performance of the operation.

It should be carried out in cases of dislocation of the spleen, prolapsed spleens, which are irreducible or already gangrenous. It should not be carried out in cases of medullary carcinoma of the spleen, as it is generally secondary to like diseases of the liver and stomach. Echinococcus of the spleen must be treated by other methods (puncture, puncture with aspiration, etc., etc.) In scrofulous and tuberculous patients, and those having diseased spleens, as a result of malaria, cirrhosis of the liver, and leukaemia, all attempts at an operation must be desisted from, and the primary affections treated in the usual manner.

It may not be out of place to make mention here of two extirpations performed in California, which have escaped the observation of Dr. Zesas. They will increase the statistics of extirpation of the spleen, and are, besides, noteworthy, from the fact that in both cases the enlarged spleen was due to malarial disease. The first case was operated upon at St. Mary's Hospital, San Francisco, by Prof. L. C. Lane. The patient had suffered from malarial fever for a long time, and had an enormously enlarged spleen. The incision was made in the linea alba, and the spleen separated from its adhesions, which were very extensive and numerous, and excised. It became necessary to open the wound after closing it, in order to control hemorrhage, which had set in.

The patient sank rapidly; transfusion was resorted to, and he rallied for a short time, sinking again and dying shortly after the operation.

The second case is one performed by Dr. J. R. Simmons, of Sacramento. Patient had also been suffering from some malarial disease. All remedies, including injections of ergotin, had been tried, but proved of no avail, and it was resolved to remove the enlarged spleen. Incision made at edge of linea alba, and spleen extirpated. Measured 15½ in length and circumference, and weighed 7½ lbs. The patient died two and a half hours after the operation, from hemorrhage.

The Medical Times and Gazette, of March 24, 1882, contains the report of another splenotomy performed by Howard. The patient was strong, and not anæmic; had never suffered from intermittent fever, presented no signs of leukæmia, except enlarged spleen. Incision in linea alba, great hemorrhage, stopped by ligature and pressure with sponges. Five hours after the operation, collapse and death. Simple hypertrophy of the spleen. Cause of death believed to be due to disturbance of cœliac plexus of sympathetic nerve.—*Dr. T. F. Morse, in Western Lancet. Detroit Clinic.*

**Placental Delivery.**—In a very large number of cases of delivery, involuntary contraction of the walls of the womb ceases after the birth of the child. These are the cases we are considering. This cessation of contraction, if the patient is left quite to herself, may last ten, twenty, forty minutes, or an hour, five or ten hours, or longer. We have been instructed to *wait*—time indefinite. And if you choose to wait, you may perhaps have to do it but for a few moments, and perhaps for a time as indefinite as the instruction. There is neither necessity nor good in waiting, except in an occasional case where your patient may be so exhausted by her previous efforts as to be quite incapable of making any more; in which condition let her have a brief rest. Do not hurry matters; but, when you have gotten the child out of your way, straighten your patient out so as to bring the abdominal muscles as closely down upon the uterus as practicable. With a bare hand on the bare abdomen, get around and hold of the fundus; take the cord in your other hand, well up, twisting it around your fingers to keep it from slipping; put it enough on the stretch to reduce the diameter of the placenta at the os and to bring the depending part into it; and say to your patient, gently, but as if you expected the proper response from her, "Come, now, give us one more pain and we will be through," or words of like import. She will fill her lungs and, beginning to "bear down," will *originate* a uterine contraction, a voluntary contraction, very readily perceived, and by which, aided by slight traction upon the cord and grasping pressure upon the uterus, the placenta will be driven out—not expressed, but expelled; and *that* is the thing desirable. If I have not been deceived by the observations of a thirty years' experience, this voluntary effort has vastly more of virtue and common sense in it than can be found in the vaunted method of Crede, or any other thing similar. A good many years ago (it was while *waiting* on a case for "a pain" to expel the placenta) the woman said, "See; let me blow on the back of my hand;" whereupon, inflating the lungs to a full capacity, she began to "bear down" while blowing on her hand, the lips being pressed to it so that no air could escape; and directly the after-birth slid into the vagina. In her mind she attributed the result to the blowing on her hand; but, as a matter of fact, she had instituted a voluntary contraction of the muscular walls of the uterus, and that had driven out the secundines. I have since seen other women "blow on the back of the hand" with the same efficacy.

It is not to be understood that a single voluntary effort of this sort will always procure the contraction, but that it often will. It is not to be understood that the effort, or repeated efforts, will not sometimes quite fail, but that they will frequently succeed. It is not to be understood that, the contraction procured, the placenta will always be cast out by the first closing in of the walls any more than by the first inclosing from an involuntary pain; but that one, two or more of these solicited and induced contractions will generally result as desired, and that the procedure indicated is one of the most potent factors in the attainment of that desired result. In a case of adherent placenta this stimulated plan is of great service. The work performed by it in separating the adhesion is precisely the same as performed by an involuntary contraction, with this in its favor, that whereas you might wait a long while for the involuntary pain to come, you can often abridge much time by the procured inclosing of the uterine structure. In cases of retained placenta after abortions, which sometimes give the practitioner a deal of trouble, the severe "bearing down" effort upon the part of the patient will often materially aid in the removal of the secondary mass.

I am aware that the power of voluntary contraction of the uterus is a matter not only called in question, but by some strenuously denied. I do not propose entering into the discussion of it here, but when, under the conditions we have been considering, one has seen the thing many times, he may be justified in believing that he knows exactly what he is talking about.—*Dr. Griswold, in Louisville Med. News.*

**Cataract, Enucleation and Iridectomy.**—In a review of recent works on the Eye, in the Archives of Medicine, the following passages occur:

The very important subject of iritis discloses little discrepancy of opinion except in the treatment of serous iritis, which Noyes does not make essentially different from that of the plastic form, while Williams says of it that "atropia is by no means to be regarded as the treatment, *par excellence*, to be used early and often as in the plastic variety." The latter considers a solution of the strength of two grains of atropia sulph. to ℥ i of water sufficiently strong to rupture adhesions; while the former advises at least double that strength, and in New York even eight grains to the ounce is frequently used.

The chapters on cataract have an especial interest by reason of the debatable points upon which we are constantly seeking the results arrived at by our most experieneed ophthalmologists.

Noyes would operate on *traumatic* cataract in one eye because of "gain in enlarging the field of vision, in the stronger mental impression, and because, notwithstanding no correcting glass was worn, a degree of stereoscopic vision was secured which the patients many times found of great advantage." Williams would not operate "because the eye operated on, having no accommodation, does not harmonize with the more perfect visual act in the opposite eye, and at times confuses instead of aiding it."

The latter would operate on both eyes at once in senile cataract, if both were ready; the former would not, preferring to give the second eye the advantage gained from the experience of the first operation. Anesthesia by sulphuric ether is recommended in most cases by Williams with a fervor inspired by a sight of the statue in the public gardens commemorating an event alluded to by all Bostonians who write medical books. Noyes says the employment of anesthesia "depends on the habit of the operator and the wishes of the patient." The use of corneal sutures to close the wound after the extraction of the lens, which was first proposed about ten years ago by Williams, is still recommended by him, though it is certainly not in general use among ophthalmologists. Removal of soft lens matter by aspiration is condemned by both authorities.

As regards the best method of extraction for the majority of cases, we find Williams rather non-committal. According to Noyes, the modern operation known as Graefe's modified linear extraction has quite supplanted the "flap method," while Williams evidently has a fond affection for the "flap method," which he performed so skilfully long before the new method was devised, and he draws attention to the fact that the development of secondary consequences—viz., separation of the retina, and irido-cyclitis—is beginning to be noticed in an alarmingly large number of cases.

The new operation of optico-ciliary neurotomy as a substitute for enucleation is more enthusiastically mentioned by Williams, who says, "there appears to be every reason for believing that it may replace the graver operation (enucleation) in a large number of the cases, at least, where the enucleation has been performed as a preventive measure, before the second eye has become affected." Noyes says, "it has claims to consideration and is being extensively tested."

Of sclerotomy, the proposed substitute for iridectomy in glaucoma, Williams says: "It seems to be conceded that though sclerotomy offers a fair chance of success, iridectomy is most to be relied on for a certain and permanent curative effect, and it is, perhaps, sometimes useful for the relief of persistent pain in absolute glaucoma, where vision is hopelessly lost." Noyes says, "My own experience with it is small, and leaves me to favor iridectomy in critical cases. The efficacy of the proceeding is not fully established, although some have written strongly in its favor."

In the treatment of stricture of the lachrymal passage there is a decided difference of opinion, Noyes favoring thorough incision at once, followed by the use of large probes, even as large as 4 mm. in some cases. Williams, on the contrary, incises very cautiously and uses probes of moderate size only.—*Pacific Med. Jour.*

**Practical Notes on Neuralgia and Its Treatment.**—There exists no better established nor more important fact than that neuralgia is a disease arising when the body is in a state of general debility. This is now more generally recognized than formerly, when pain was too often regarded as the symptoms of what was termed "sthenic inflammation" to be energetically treated by low diet and repleting remedies.

As this disease is frequently mistaken for rheumatism, gout, spinal irritation, etc., and *vice versa*, it may be well to name some of the leading features of a typical case of neuralgia. 1. It occurs when general debility exists, is increased by fatigue, mental or bodily, but relieved by food and sometimes by stimulants. 2. The pain, which is sudden, darting and excruciating, exhibits remarkable intermissions, especially in the early stages of the complaint, and the constitutional disturbance is slight (temperature, pulse, etc., frequently normal.) 3. It is usually unilateral. 4. As the disease advances tender spots (points douleureux) are formed in the course of the affected nerves.

That debility is a prime factor in neuralgia we have but to call to our remembrance cases which constantly appear. The overworked, anæmic, badly fed girl, suffering from neuralgia of the fifth, the anxious, struggling man in the early years of professional life or business, the married woman weakened by child bearing or over zealous in domestic cares, and the neuralgia of declining years, degeneration having set in, nutrition being defective. In our diagnosis we are assisted by the family history of the case, whether nervous disease in any of its varied forms has existed.

The treatment should be directed in every case toward improving the general health. Nutrition must be improved by very nourishing food, well masticated, and if stimulants are prescribed they should be given with food; pure air night and day; great cleanliness and the use of sponging with sea salt and water. Cod liver oil and cream are of service, given after meals. Quinine in facial neuralgias, and also chloride of ammonium; arsenic in cases of angina pectoris; iron and strychnine in anæmic states. Bromide of potassium is useful in mild cases, where the pain is not severe, but a general nervous condition exists, with restless irritability. The subcutaneous injection of morphia, beginning with one-sixth of a grain, is the most speedy and useful remedy we possess, and is a curative agent; for it checks at once pain, and thus gives us the opportunity of carrying out all those constitutional measures for improving the general health, whilst it disturbs but little appetite and digestion, and with use a toleration is established, and appetite sometimes improved; for nothing is more apt to destroy appetite than the distress of severe pain. In chronic cases of neuralgia, a blister, not necessarily carried to the point of vesication, is often of the greatest possible service, and it is a treatment peculiarly adapted to old standing intractable cases.

Having sketched the mode of treatment it is unnecessary to give illustrations of the ordinary cases which constantly present themselves in hospital and private practice. I therefore select from my note book one of several successful cases where neuralgia has occurred in that period of life when a cure is rarely accomplished (some authorities say never)—the degenerative period.

In March, 1877, I saw, in consultation with Dr. Walker, of Wakefield, a lady aged seventy-six, who in early life had suffered from neuralgia of the stomach, which had been much aggravated by the treatment then in vogue of insufficient nutritive food and depleting remedies. This patient was seized with violent pain, affecting the nerves of the scalp, and which became so excruciating as to deprive her of sleep for many successive nights. She became delirious in consequence, and we decided to inject one-quarter of a grain or morphia. This gave prompt relief and procured sleep. She was ordered turtle soup, oysters, and an exceedingly nutritious dietary. She was well supplied with food at night also, which invariably relieved the pain. A mixture, containing half-drachm doses of aromatic spirit of ammonia and fifteen minims of tincture of nux vomica, seemed greatly to improve the appetite, which became prodigious and surprising. The tendency to degenerate was kept prominently in view, pure air was freely supplied in the bedroom, and every other measure taken to improve nutrition and the general health. As a local application, the chloroform liniment with tincture of opium relieved pain, and as soon as the case became chronic, the hair was cut closely and blistering fluid applied to the tender spots, which were well developed in this case; multiple abscesses formed, and were frequently opened by Dr. Walker. The old lady, after an illness of three months' severe suffering, recovered perfectly, left Wakefield for Harrogate, and is now (1882) in fair health, having had no return whatever of her former complaint. Her body is feeble, but her mind extraordinarily clear and bright for a lady who has passed her eighty-first year.—*London Lancet.*

**A New Sleep-producing Agent.**—Professor C. Binz, in a series of articles contributed to the Berliner klinische Wochenschrift, announces the discovery of nerve-depressing and sleep-producing properties in ozone.

The accepted view regarding this gas has been that it is very easily decomposed, nascent oxygen being set free; that it is extremely irritating on this account to the tissues, acting much like chlorine, and that it cannot be absorbed by the blood. Binz, however, shows that, in proper quantities, it is not irritating, can be inhaled and absorbed, producing, as he claims, peculiar effects on the nervous system.

The gas was generated by the sparks of an electric battery containing four of Bunsen's element. The ozonized air was conducted by a tube through chloride of calcium. It was then carried by a tube either to a large air-tight glass bell, in which an animal was placed, or to a mask which was worn by the persons who inhaled it. Animals were first tried. If a strong and long-continued dose of the ozone was supplied, the usual symptoms of laryngeal and tracheal catarrh with strangulation and death occurred. If supplied in more diluted quantities for less than two hours, sleep or a lethargic condition was produced. Frogs, rabbits, and kittens, reacted best. The latter would, in the course of ten or fifteen minutes, become quiet and then lie down and apparently sleep. Shaking the jar would not arouse them. When removed and supplied with fresh air, however, they soon returned to their normal condition. Several animals were killed after having been in this condition, and no changes in the air-passages or other tissues noted. Precautions were taken and experiments made to show that there was no carbonie acid-poisoning and no introduction of nitrous oxide gas. The animals could, as a rule, be kept in the bell jar for two hours before any symptoms of irritation appeared, even of the outer parts of the air-passages.

The experiments were then tried upon human beings, Dr. Hugo Schultz was the first to submit himself. Subsequently five other gentlemen inhaled the gas. Three of them were put to sleep by it, the others were slightly stupefied or otherwise depressed. The time required for bringing on sleep varied between six and sixteen minutes. The sensations during this time were very agreeable. After removal of the gas the sleeper would awake within half a minute, generally sooner. It was suggested that in one quite susceptible person the condition was a hypnotic one, but inhalation in the same way of pure air produced no effect. After awaking there was some feeling of fatigue, but this soon passed away.

Large and prolonged doses of the gas produced sensations of nausea, dizziness, and strangling. But the diluted ozone was breathed for over half an hour without harm. Binz states that in too small amounts no effect is gotten; in too large ones, irritation is produced. He compares its action in this respect to that of alcohol when given. Prof. Binz claims no practical results from his discovery as it stands at present, but thinks that like every new scientific truth it may have, eventually, some useful bearing.—*N. Y. Med. Record.*

**Nitrous Oxide.**—Further experience has not changed the relative position or very much enlarged the sphere of action of nitrous oxide. That it is the safest of all anæsthetics has been established beyond a question. In one institution where such administration is subject of record, this gas has been given over 100,000 times, and not only without a death but without causing in a single instance symptoms sufficiently serious to necessitate transporting the patient home in a carriage. In the city of Philadelphia alone, it has been given over 133,000 times without a death, and without any injurious results. Death cannot be justly attributed to it in more than four cases since its introduction.—*F. C. Reeve, in Holmes' Surg., Amer. Edition.—Med. Rec.*

**Treatment of Diabetes by Bromide of Potassium.**—Before the meeting of the Academie de Medicine a member read a paper on the treatment of diabetes by bromide of potassium. For the last six years the author has made this disease the object of his researches, and during that period he treated fifteen cases. He ignored entirely the classic *regime* of gluten bread, etc., being of the opinion that the disease consisted, not in the presence of sugar in the urine, but in the disorder of the organism, which produced the sugar in excess. Having had a patient who was diabetic, but who consulted him for certain nervous affections, he observed that under the influence of the bromide of potassium, of which he prescribed a drachm a day, the former disease yielded. This case gave him the idea to make experiments on rabbits, in which he produced artificially diabetes in touching the flour of the fourth ventricle, according to the method of Claude Bernard. Four grains of the drug injected into the veins caused the sugar to disappear in each case. Consequently, ever since, the author has entirely adopted this drug in the treatment of the disease in question, and always with good results. The author further insisted on the necessity of employing muscular exercise of every kind. The use of alkalies, and of iron, arsenic, quinine, according to their several indications, form part of the general treatment. There is one point worthy of remark in the above communication, and that is the complete disregard as to *regime*, which must be beneficial to the patient in that he is relieved from the irksomeness of observing a certain imposed diet, which soon disgusts him, and what is more grave, keeps him continually dwelling on the affection from which he suffers, a fact which often leads to another malady, which, though less formidable, is not without producing a deleterious effect on the constitution, already weakened by the primitive disease, hypochondria. If further researches verify the efficacy of the observations of the learned author, the treatment of this grave affection will have made a *pas en avant.—Medical Press, August 30, 1882.*

**Recovery of Nine Cases of Hydrophobia.**—At a recent meeting of the Paris Academy of Medicine a memorandum was read by M. Decroix, reporting nine cases of cure of hydrophobia. The Committee on Rabies made, during the year 1874, a series of experiments with medicines said to be useful for curing rabies, in which they made use of pilocarpin three times, and in every case the remedies hastened death by the violent fits they brought on. In the course of his experience, M. Decroix met with two cases of rabies which did not end fatally. The conclusions arrived at by the Committee are as follows:

First.—It has been experimentally demonstrated that rabies may recover spontaneously.

Second.—Up to the present no treatment has proved to be antihydrophobic, and cases of cure by this or that means may be attributed to the efforts of nature.

Third.—All the means used by the Committee since 1874, comprising principally injections of pilocarpin, have hastened rather than retarded the death of the subject.

Fourth.—Those dogs usually recovered which were left without treatment, as the medicines brought on violent fits, and there is an inclination among medical men to leave men thus attacked in perfect quiet, and only practice experiments on animals The filing down of dogs' teeth—an easy and almost painless operation—is still the most efficacious preventive of madness.

Fifth.—Rabid people left in the dark and kept quiet are not subject to fits, unless they are brought on by excitement or by ordinary medicines, and "as far as I am concerned," says M. Decroix, "I would rather be attacked by this kind of madness than many other diseases, particularly than that red chancre of smokers."—*Medical Press.*

**The Treatment of Intussusception.**—In the September number of the New York Medical Journal and Obstetrical Review, Dr. W. R. Gillette, physician at Bellevue Hospital, relates a case of intussusception in a child nine months old, relieved by injections of water, the administration of chloroform by inhalation, and manipulation of the tumor felt through the abdominal wall. This, he states, is the third case of intussusception in infants which he has seen, and which he has been able to reduce by these means. He thinks that these cases, from the philosophy of their condition, and the necessary measures for relief, are best managed in the way indicated In two other instances, in which he saw and advised this treatment, reduction was utterly impossible under the other methods tried. The children in each of these cases were held while struggling, and the injections forced into them against all voluntary and involuntary efforts which they could make. He deems the administration of chloroform almost absolutely necessary in these cases. The reason is not difficult to find, inasmuch as, while it gives us such perfect control of the patient, it also eliminates the element of muscular spasm. Moreover, massage is a powerful adjuvant to the hydrostatic pressure of water in these cases. In the first two cases the obstruction was not overcome until massage also was employed.—*Ind. Prac.*

**Treatment of Yellow Fever by Phenic Acid.**—M. Lecaille, of Rio Janeiro, following out the doctrine of ferments, has been treating yellow fever with phenic acid. In twelve cases under his care, success was complete. In one instance the stage of "black vomit," etc.. had set in, and the patient was almost moribund. Lecaille saw him at this time, *i. e.*, the fifth day. Phenic acid was injected hypodermically, syrup of the phenate of ammonium was given per orem, and sulpho-phenic per rectum. This was done at intervals of two hours. On the third day of treatment the patient was pronounced cured. A second patient was in the cosmic stage of the parasite. Ten hypodermics, together with rectal injections of sulpho-phenic and glyco-phenic were made, with the result of bringing about a cure on the seventh day.—*Cronica Med. Quir.*

**Viability of Premature Children.**—Professor Spath recently expressed himself as a believer in the very early viability of premature children (Wien. Med. Zeitung, May 20, 1882). After speaking of the generally accepted opinion that children were not to be considered viable until after the completion of seven lunar months gestation—twenty-eight weeks (the Prussian law pronounces the fœtus viable that has been carried thirty weeks)—he went on to say that his own observations warranted him in making the statement that a fœtus could be kept alive out of the sixth month even. Of course, the sixth lunar month is from the end of the twentieth to the end of the twenty-fourth week of utero-gestation. Particular care would naturally be demanded in bringing up such youngsters. Enveloping the child in cotton wadding was an admirable way of preventing loss of the low degree of body-heat such children possessed. The weakness of the digestive powers should be met by giving such milk as contains but a small amount of casein. As observation shows that the milk of women becomes richer in casein the longer they suckle, only young girls should be made choice of as wet-nurses, who have been recently confined. It was also necessary that the young person chosen should have long nipples; for, as the infant itself would be too weak to suck and swallow, the nipples ought to project deeply into the mouth, so that the milk might in a manner run of itself into the stomach. Professor Spath himself had a case under observation in which a child born *in* the sixth lunar month was successfully brought up in the manner above stated, the child being at the present time six years old, and quite as well grown and strong as its brothers and sisters born at full term. He remarked, in conclusion, that the great mortality of premature children was easily comprehended when one bore in mind the ordinary high death-rate during the first year of infant life.—*Med. Press and Circular.*

**Poisoning from Red Stockings.**—Dr. J. Woodward, in The Lancet, calls attention to the fact that an irritation of the feet and legs, followed by small pustules and a subsequent exfoliation is sometimes caused by red stockings. Upon a careful analysis of some of the stockings he found that a tin salt, which had been used as a mordant in fixing the dye, was present in considerable quantity. He succeeded in obtaining as much as 22.3 grains of this metal in the form of the dioxide, and as each time the articles are washed the tin salt is rendered more easily soluble, the acid excretions from the feet attack the tin oxide, thus forming an irritating fluid.—*Louisville Med. News.*

**Geum Album for Gastric Irritation and Headache.**—Dr. W. A. Spurgeon, in Therapeutic Gazette, March, 1882, says, after a botanical description of this plant, that it is already useful as an anti-emetic; that it relieves gastric irritation (from any cause) and headache. A teaspoonful of a tincture, representing eight troy ounces to the pint, is a dose, but larger doses may be given.—*Virginia Med. Monthly.*

**Pet Animals and Contagious Diseases.**—Dr. Hewitt, of Lake Superior, relates in the Journal of Compar. Med., an instance in which diphtheria was communicated by a cat. For several days a pet cat had been suffering with enlarged cervical glands, other cats were similarly affected. The pet cat died in the house, and on the day of its removal, there broke out in his family a most virulent form of diphtheria, resulting in the death of two of his children, the doctor barely escaping with his life. Up to this time the neighborhood was remarkably free from sickness of any kind. The disease spread, and very soon a large portion of the inhabitants were down with the disease.

It is to be hoped that more attention will be paid to this subject and instances, similar to the above, reported.—*N. Lancet.*

**Treatment of Syphilis.**—After dwelling upon the importance of exhausting every conceivable means of diagnosis, Sigmund, as the result of his long experience, advises removing the initial lesion (if the case be seen very early) with knife, cautery, or caustic, followed by neat dry dressings. After this he advises deferring constitutional treatment, except hygienic, until the cutaneous manifestations appear. When this arrives he uses for the lighter forms the iodine preparations; for graver forms with defective nutrition and strength, palpably due to syphilis alone or widespread pustular, papular or squamous eruptions, mercury. But this must never be pushed to salivation. For the gravest tertiary forms he recommends mercury and iodides alternately.—(*Neuere Behandlungsweise der Syphilis.*)—*Amer. Prac.*

**Small-Pox and Scarlatina Occurring Simultaneously in Same Subject.**—Chrostowski (Gaz. Lekars., 1881, No. 53) relates a case in a patient æt. 19. On the fifth day after scarlatina had been diagnosed, red spots and papules appeared over the face; the upper limbs, hard palate and other parts remaining free from the scarlatinous rash. Temperature immediately fell from 105.5° to 103.6°. For some days new papules continued to appear, always selecting with mathematical preciseness non-scarlatinous spots. The change of papules into pustules coincided with desquamation of the scarlatina eruption over the trunk. After, the course of the small-pox was regular and generally mild. Four weeks later the patient left cured. No albuminuria.—*Lond. Med. Record, July 15, 1882.*

**Small-Pox in Birds.**—Dr. Hewson, of Philadelphia, claims that he has traced this disease to the English sparrows' nests. The senior editor of the Pittsburgh Medical Journal has seen the eruption of small-pox among the poultry of a family he was attending for that disease, in 1849. The disease was manifested principally upon the head and comb of the fowl, and the parts beneath the bill not covered with feathers. These parts were covered with pustules resembling those met with in the human subject, closing the eyes and swelling the head to double its former size. The disease appeared to be contagious, and was quite fatal.—*St. Louis Clin. Record.*

